# *PNPLA3* rs738409 G allele carriers with genotype 1b HCV cirrhosis have lower viral load but develop liver failure at younger age

**DOI:** 10.1371/journal.pone.0222609

**Published:** 2019-09-17

**Authors:** Renata Senkerikova, Sona Frankova, Milan Jirsa, Miluse Kreidlova, Dusan Merta, Magdalena Neroldova, Klara Chmelova, Julius Spicak, Jan Sperl

**Affiliations:** 1 Department of Hepatogastroenterology, Transplant Centre, Institute for Clinical and Experimental Medicine, Prague, Czech Republic; 2 Charles University, First Faculty of Medicine, Prague, Czech Republic; 3 Laboratory of Experimental Hepatology, Institute for Clinical and Experimental Medicine, Prague, Czech Republic; 4 Institute of Medical Biochemistry and Laboratory Diagnostics First Faculty of Medicine Charles University and General University Hospital in Prague, Prague, Czech Republic; 5 Anesthesiology, Resuscitation and Intensive Care Department, Institute for Clinical and Experimental Medicine, Prague, Czech Republic; Medizinische Fakultat der RWTH Aachen, GERMANY

## Abstract

**Background:**

*PNPLA3* rs738409 minor allele c.444G represents a risk factor for liver steatosis and fibrosis progression also in chronic hepatitis C (HCV). We investigated its impact on the timing of liver transplantation (LT) in patients with genotype 1b HCV cirrhosis.

**Methods:**

We genotyped and evaluated 172 LT candidates with liver cirrhosis owing to chronic HCV infection, genotype 1b. One hundred patients needed LT for chronic liver failure (CLF) and 72 for a small hepatocellular carcinoma (HCC) in the cirrhotic liver without CLF. Population controls (n = 647) were selected from the Czech cross-sectional study MONICA.

**Results:**

The CLF patients were younger (53.5 ± 7.2 vs. 59.6 ± 6.6, *P* < 0.001) with more advanced liver disease than HCC patients (Child-Pugh’s score 9.1 ± 1.8 vs. 7.1 ± 1.9, *P* < 0.001, MELD 14.1 ± 3.9 vs. 11.1 ± 3.7, *P* < 0.001). *PNPLA3* G allele increased the risk of LT for CLF in both allelic and recessive models (CG + GG vs. CC: OR, 1.90; 95% CI, 1.017–3.472, *P* = 0.045 and GG vs. CC + CG: OR, 2.94; 95% CI, 1.032–7.513, *P* = 0.042). Multivariate analysis identified younger age (*P* < 0.001) and the G allele (*P* < 0.05) as risk factors for CLF. The genotype frequencies between the CLF group and MONICA study significantly differed in both, allelic and recessive model (*P* = 0.004, OR 1.87, 95% CI 1.222–2.875; *P* < 0.001, OR 3.33, 95% CI 1.824–6.084, respectively). The OR values almost doubled in the recessive model compared with the allelic model suggesting the additive effect of allele G. In contrast, genotype frequencies in the HCC group were similar to the MONICA study in both models. Pretransplant viral load was significantly lower in GG than in CC + CG genotypes (median, IQR; 162,500 (61,550–319,000) IU/ml vs. 570,000 (172,000–1,595,000) IU/ml, *P* < 0.0009).

**Conclusions:**

Our results suggest that *PNPLA3* rs738409 G allele carriage may be associated with a faster progression of HCV cirrhosis to chronic liver failure.

## Introduction

Adequate timing of liver transplantation (LT) represents one of the main factors determining favourable posttransplant outcome. Prediction of the patients’ prognosis based on the known natural course of a particular liver disease is crucial in the evaluation process [1]. The natural course of liver diseases may be altered by genetic factors. Single nucleotide polymorphism rs738409 c.444C>G (p.Ile148Met) in the patatin-like phospholipase domain-containing protein 3 (*PNPLA3*) is nowadays one of the most important genetic factors with an impact on progression of several liver diseases of different etiology [2].

Association between liver fat content as a quantitative trait and *PNPLA3* rs738409 genotype was described in a large genome-wide association mapping study [3] in 2008 and confirmed in a more detailed study [4] by the same group of authors in 2014. More than fifty studies demonstrating that the *PNPLA3* rs738409 G allele is a risk factor for non-alcoholic steatohepatitis (NASH), liver cirrhosis in NASH or alcoholic liver disease have been published in the past decade [5–11]. The same allele was also identified as a risk factor for liver fibrosis and cirrhosis in HCV-monoinfected individuals [12–16] and in those with HCV/HIV coinfection [17–20] and it also turned out to be a predisposing factor of hepatocellular carcinoma (HCC) [21–24]. In a recent study, the increased risk of HCC and *PNPLA3* G allele was found only in alcoholic liver disease, but not in non-alcoholic fatty liver disease or viral hepatitis B and C [25].

Whereas the impact of the G allele on the liver fibrosis progression in chronic hepatitis C seems to be well known, its impact on chronic liver failure (CLF) progression and the need of LT has not been described so far. In this study, we aimed to investigate the impact of *PNPLA3* genotypes on the risk of CLF in a homogenous group of cirrhotic patients infected with HCV genotype 1b.

## Patients and methods

### Study design and eligibility of patients

We retrospectively evaluated 172 adult patients with HCV-related cirrhosis caused by HCV genotype 1b with Child-Pugh’s class A, B and C who underwent LT between January 1995 and August 2018 at our center. One hundred patients were enlisted for LT and transplanted for CLF (CLF group) using standard criteria evaluating liver dysfunction according to the Child-Pugh’s and MELD score and 72 patients were transplanted for a small HCC (HCC group). Fifty-two patients fulfilled Milan criteria, remaining 20 complied with San Francisco or up-to-seven criteria based on pre-transplant imaging techniques results [26–28]. The diagnosis of HCC was confirmed in the liver explants using standard histological staining techniques. Neither patients with HBsAg positivity nor those with HBcAb positivity were included. Patients combining HCV infection with excessive alcohol consumption (60 g per day in males and 40 g per day in females) were also excluded. None of HCV-infected patients had obtained antiviral treatment in the year preceding LT in accordance with our centre anti-HCV treatment policy: very short times in the liver transplant waiting list, 80–90 days, do not allow for a safe entire treatment course before LT, even in the era of direct acting antivirals. The patients were treated after LT according to the period of transplantation, using an interferon-based regimen until 2014 or a direct acting antivirals combination thereafter. Demographic, clinical, laboratory and histological data were collected from the internal hospital and outpatient database (S1 Table).

Genotype frequencies in both CLF and HCC groups were compared with the *PNPLA3* genotype frequencies in 647 subjects 0.566/0.372/0.062 (CC/CG/GG) reported in the Czech cross-sectional population study MONICA [29], genotyping data were taken from Trunecka et al. [30].

### HCV viral load and HCV genotype assessment

HCV viral loads (serum HCV RNA levels) were determined in blood samples taken from HCV-infected patients within 24 hours before LT (last value unaffected by immunosuppression or antiviral therapy). In 133 patients, serum HCV RNA level was assessed according to the period of sampling by the Roche COBAS^®^ AmpliPrep/COBAS^®^ TaqMan^®^ HCV Quantitative Test v1.0 or v2.0 (Roche Molecular Systems Inc., South Branchburg, NJ).

In the 39 remaining patients, only an in-house quantitative method was used and therefore those results were not included in the statistical analysis. HCV genotype was assessed using the SIEMENS Versant^®^ HCV Genotype 2.0 Assay (LiPA) (Siemens Healthcare Diagnostics Inc., Tarrytown, NY).

### Genotyping

DNA was isolated from the peripheral blood using the Qiagen QIAamp kit (Qiagen, Hilden, Germany). All patients were genotyped for the *PNPLA3* rs738409 c.444C>G polymorphism by the TaqMan predesigned SNP genotyping assay No. C_7241_10 (Thermo Fisher Scientific, Waltham, MA). Genotyping was performed according to the manufacturer’s protocol using the Applied Biosystems ABI 7300 Real-Time PCR instrument (Thermo Fischer Scientific). No significant deviation from the Hardy-Weinberg equilibrium was observed in *PNPLA3* genotypes distribution within the CLF and HCC patient groups.

### Statistical analysis

Continuous variables are presented as means and standard deviations, whereas categorical variables are expressed as frequencies (%). Categorical data were analyzed using the chi-square test. For continuous data, Student’s t-test or the non-parametric Mann-Whitney test were used appropriately. Genotype frequencies were determined and tested for consistency with the Hardy-Weinberg equilibrium using the chi-square test. Testing for genetic associations was performed as described in [31]. Risk factors were examined using multivariate logistic regression analysis. All statistical analyses were two-sided and *P* value of < 0.05 was considered statistically significant throughout the study. Statistical analysis was performed using the R programming language version 3.2.0 (www.r-project.org).

### Ethics statement

This study was approved by the Ethics Committee of the Thomayer Hospital and Institute for Clinical and Experimental Medicine, Prague, Czech Republic, and was carried out in compliance with the Helsinki Declaration. The patients’ informed consent was not required by local law because of the retrospective design of the study and the use of data from which the patients’ identification information had been removed. All study participants gave written consent to the storage of blood samples and agreed to using blood for future research including genetic testing. The written consent was obtained before enlistment for LT.

## Results

### Demographic, clinical data and laboratory data

Demographic, clinical and laboratory data of the CLF and HCC groups are shown in Table 1. Patients transplanted for CLF were younger with a higher proportion of males and suffered from more advanced liver disease according to the Child-Pugh’s and MELD score in comparison with the HCC group. Patients in CLF group had lower AFP levels and lower total cholesterol, HDL and serum triglycerides levels.

**Table 1:** Demographic, clinical and laboratory data of subgroups with CLF and HCC.

Variables	CLF group n = 100	HCC group n = 72	*P* value
Males (n)	68 (68.0%)	38 (52.8%)	0.0428
Age (years)	53.5 ± 7.2	59.6 ± 6.6	< 0.001
BMI (kg/m^2^)	26.2 ± 4.2	26.8 ± 3.7	0.175
Type 2 diabetes mellitus	27 (27.0)	25 (34.7)	0.277
Child-Pugh’s class			< 0.001
A	6 (6.0)	37 (51.4)	
B	48 (48.0)	27 (37.5)	
C	46 (46.0)	8 (11.1)	
Child-Pugh’s score (points)	9.1 ± 1.8	7.1 ± 1.9	< 0.001
MELD score (points)	14.1 ± 3.9	11.1 ± 3.7	< 0.001
Ascites			< 0.001
None	44 (44.0)	53 (73.6)	
Small	32 (32.0)	14 (19.5)	
Large	24 (24.0)	5 (6.9)	
AFP (μg/l)	34.5 ± 50.1	337.1 ± 926.8	< 0.001
Total bilirubin (μmol/l)	51.8 ± 77.3	35.6 ± 46.7	< 0.001
Albumin (g/l)	29.0 ± 6.5	33.5 ± 6.8	< 0.001
ALT (μkat/l)	1.3 ± 0.9	1.5 ± 1.2	0.117
Total cholesterol (mmol/l)	3.4 ± 1.0	3.7 ± 1.0	0.004
HDL cholesterol (mmol/l)	0.9 ± 0.4	1.1 ± 0.4	0.037
LDL cholesterol (mmol/l)	1.9 ± 0.8	2.1 ± 0.7	0.080
Triglycerides (mmol/l)	1.1 ± 0.5	1.3 ± 0.7	0.009
Prothrombin time (INR)	1.4 ± 0.3	1.2 ± 0.2	< 0.001

Data are given as number, number (%), or mean ± SD.

Abbreviations: CLF, chronic liver failure; HCC, hepatocellular carcinoma; BMI, body mass index; MELD, Model for End-Stage Liver Disease; AFP, alpha-fetoprotein; ALT, alanine-aminotransferase; HDL cholesterol, high density lipoprotein cholesterol; LDL cholesterol, low density lipoprotein cholesterol; INR, International normalized ratio.

### Pretransplant viral load

Pretransplant viral load was known in 133 of 172 HCV cirrhotic patients. HCV patients with known pretransplant viral load included 66 of 82 patients with the *PNPLA3* rs738409 CC genotype, 51 of 67 patients with the CG genotype and 16 of 23 patients with the GG genotype.

**Fig 1:**
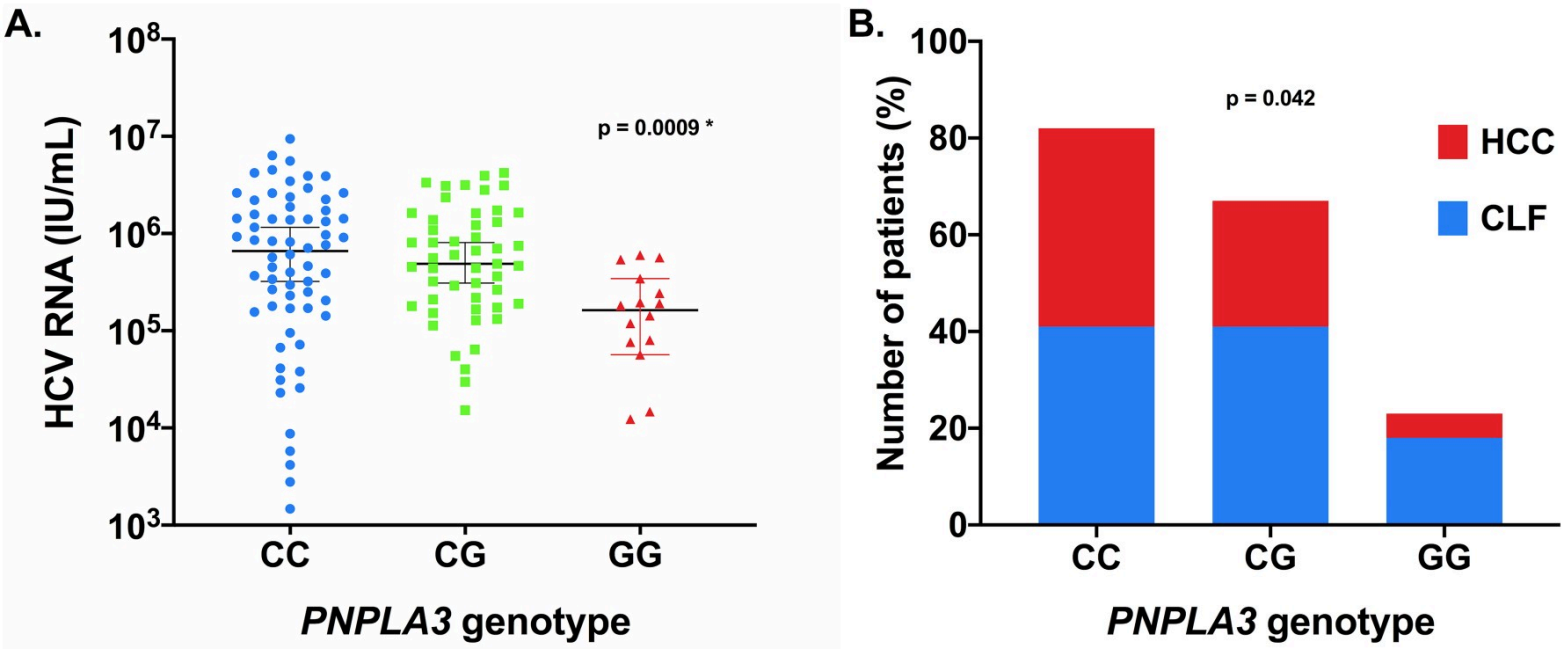
Impact of *PNPLA3* rs738409 genotypes on pre-transplant HCV RNA levels (panel 1*A*) and percentage of patients with CLF (panel 1*B*). Pre-transplant HCV viral load assessed in 133 of 172 patients. Data are given as medians and interquartile ranges. * p value for recessive model.

Similarly, pretransplant viral load was available in 68 of 100 patients with CLF and in 65 of 72 patients with HCC. *PNPLA3* GG homozygotes had a significantly lower pretransplant HCV viral load in comparison with the C allele carriers (median, interquartile range [IQR]; GG 162,500 (61,550–319,000) IU/ml vs. CC+CG 570,000 (172,000–1,595,000) IU/ml, *P* < 0.001, Fig 1*A*). Pre-transplant viral load was significantly lower in patients with CLF in comparison with patients with HCC (median [IQR]; CLF 292,500 (83,725–829,801) IU/ml vs. HCC 806,000 (237,000–1,680,000), *P* = 0.008).

### *PNPLA3* rs738409 genotype association with CLF

*PNPLA3* genotype frequency differences between the CLF and HCC groups were found in both allelic and recessive models (Table 2*A*) (p < 0.05). Genotype frequencies between the CLF group and Czech cross-sectional population study MONICA significantly differed with *P* = 0.004 for the allelic model (OR 1.87, 95% CI 1.222–2.875, test power with α = 0.05: 0.85) and *P* < 0.001 for the recessive model (OR 3.33, 95% CI 1.824–6.084 (Table 2*B*). The OR values almost doubled in the recessive model compared with the allelic model indicating the additive effect of allele G (Fig 1*B*). By contrast, genotype frequencies in the HCC group were the same as in the MONICA study in both models (Table 2*C*). Importantly, the minor allele frequency in the MONICA study (0.25) did not differ from the frequencies recorded in European population subsets of the GnomAD (0.23) and ExAC (0.23) databases [32].

**Table 2:** Genotype frequencies of *PNPLA3* rs738409 C>G polymorphism in the CLF group, HCC group and the MONICA study.

**A**	Locus	Genotype	CLF group (n = 100)	HCC group (n = 72)	OR	95% CI	*P* value
	*PNPLA3* rs738409 c.444C>G	CC	41 (41%)	41 (57%)	1	-	-
		CG	41 (41%)	26 (36%)	1.90	1.017–3.472	0.045^a^
		GG	18 (18%)	5 (7%)	2.94	1.032–7.513	0.042^b^
**B**	Locus	Genotype	CLF group (n = 100)	MONICA (n = 647)	OR	95% CI	*P* value
	*PNPLA3* rs738409 c.444C>G	CC	41 (41%)	366 (57%)	1	-	-
		CG	41 (41%)	241 (37%)	1.87	1.222–2.875	0.004^a^
		GG	18 (18%)	40 (6%)	3.33	1.824–6.084	< 0.001^b^
**C**	Locus	Genotype	HCC group (n = 72)	MONICA (n = 647)	OR	95% CI	*P* value
	*PNPLA3* rs738409 c.444C>G	CC	41 (57%)	366 (57%)	1	-	-
		CG	26 (36%)	241 (37%)	0.98	0.602–1.610	0.951^a^
		GG	5 (7%)	40 (6%)	1.13	0.432–2.968	0.800^b^

^a^ Allelic model (*PNPLA3* CG + GG vs. CC),

^b^ Recessive model (*PNPLA3* GG vs. CC + CG)

Abbreviations: CLF, chronic liver failure; HCC, hepatocellular carcinoma; MONICA, MONItoring trends and determinants in Cardiovascular disease; OR, odds ratio; CI, confidence interval

The proportion of CLF in HCV cirrhotic patients grouped according to their *PNPLA3* rs738409 genotypes is shown in Fig 1*B*.

### Risk factors for the need of liver transplantation

**Fig 2:**
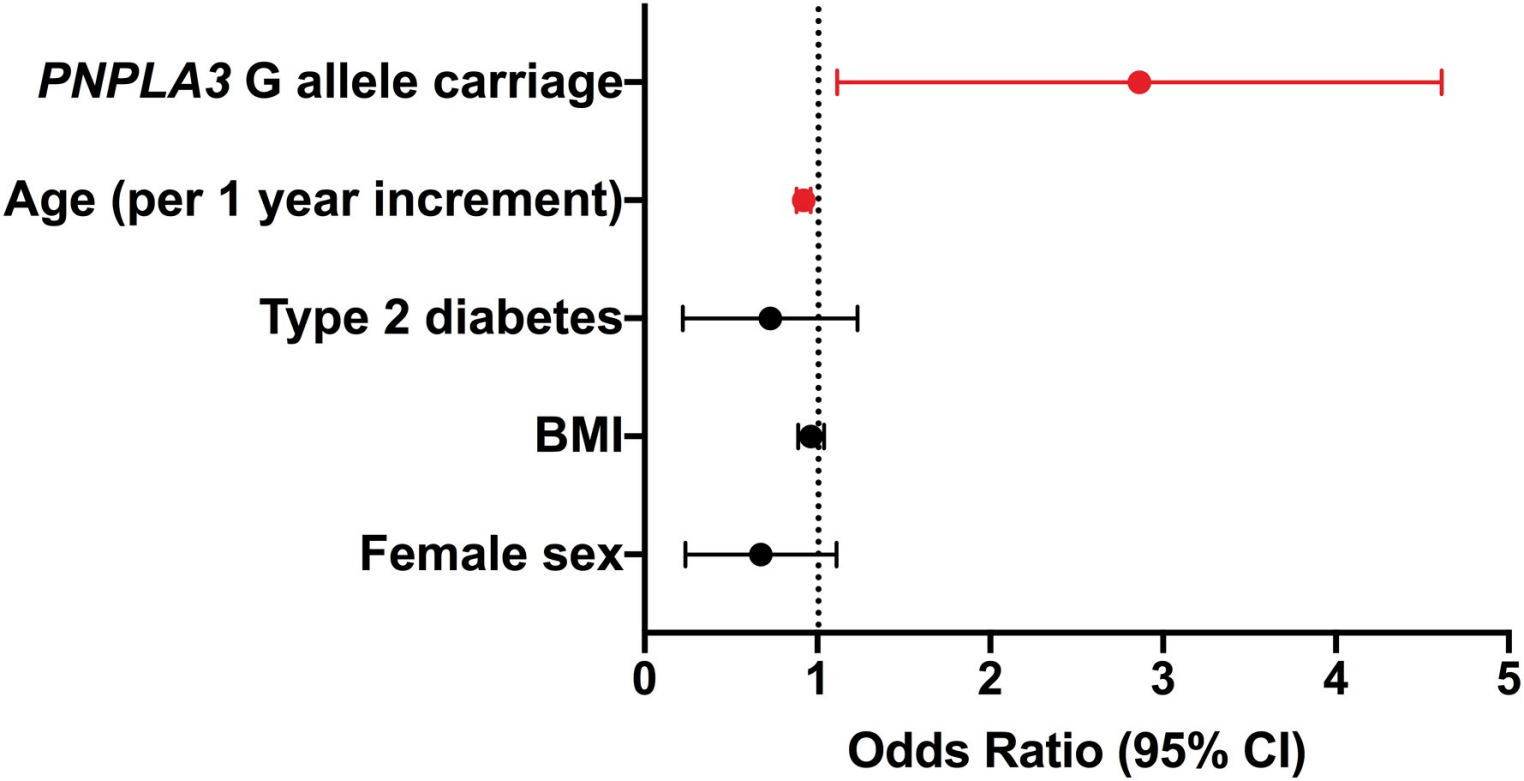
Risk factors for the need of liver transplantation: Multivariate analysis. Bars represent OR with 95% confidence interval.

In multivariate logistic regression analysis, age and *PNPLA3* rs738409 genotype turned out to be significant determinants of the need of LT. Specifically, presence of the *PNPLA3* G allele increased the risk of LT in CLF 2.4-fold (Fig 2). Other investigated variables such as gender, BMI and type 2 diabetes mellitus did not influence the risk of LT.

## Discussion

The study was prompted by our long-term observation that the liver transplant candidates with HCV genotype 1b decompensated liver cirrhosis (or CLF) had significantly more advanced liver dysfunction and were younger that liver transplant candidates with a small HCC. A similar difference in the degree of liver dysfunction between liver transplant candidates indicated for HCV with or without HCC was reported by others [33–35]. However, the age difference between liver transplant candidates was not significant probably due to the fact that the patients enrolled in these studies were infected with all HCV genotypes and HCV genotype may modify the risk of HCC [36].

To explain the age difference in our cohort, we assumed that the clinical difference between HCV liver transplant candidates with or without HCC might be caused by some genetic factor. A single nucleotide polymorphism *PNPLA3* rs738409 c.444C>G was identified as a risk factor for concurrent liver steatosis and a faster liver fibrosis progression in patients with chronic HCV infection in the past, but its impact on the need and timing of LT has not been evaluated. In our study, we identified further consequences of the carriage of the G allele: accelerated CLF development requiring LT at a younger age and lower pretransplant blood viral load. The CLF patients were younger than HCC patients and had a significantly higher frequency of *PNPLA3* allele G in comparison with HCC patients as well as with population controls.

The earlier need for LT suggests that the G allele carriage is a strong factor contributing to liver fibrosis progression. Consistently with our results, the recently published studies also presented the G allele carriage as a factor accelerating liver fibrosis progression in patients infected with chronic HCV infection. The meta-analysis by Fan and colleagues [12] showed that Caucasians with chronic HCV infection carrying the GG genotype have a more pronounced liver fibrosis and steatosis. In line with these findings, association of the GG and CG genotypes with progression of liver fibrosis was also demonstrated in a large cohort of HCV-infected patients in the HALT-C study [13].

In contrast to studies documenting association of the *PNPLA3* rs738409 genotype with the risk of HCC development in alcoholic liver disease and non-alcoholic fatty liver disease [21–23], no such association was found in HCV-infected subjects [13, 25]. This led us to initial misinterpretation of our data that the *PNPLA3* G allele was protective from HCC. However, since the G allele carriers underwent liver transplant for CLF at younger age, we realized that they were not able to develop HCC later in the course of the disease. Indeed, age is a well-known risk factor of HCC in patients with chronic HCV infection [37, 38].

As mentioned above, *PNPLA3* G allele carriers with chronic HCV infection have also more pronounced liver steatosis. We assume that in these subjects, lipid accumulation in hepatocytes with subsequent steatohepatitis accelerates progression of liver fibrosis caused by the underlying liver disease which is chronic HCV infection. Indeed, coincidence of chronic HCV infection with lipid accumulation and steatohepatitis results in more rapid development of CLF in comparison with HCV-infected individuals without steatohepatitis [12–16]. The hypothesis of two independent synergic processes leading to CLF (HCV infection and steatohepatitis) is further supported by Jimenez-Sousa et al. [15] who demonstrated a dose dependent effect of *PNPLA3* G allele on the progression of liver stiffness in HCV infected individuals. Finally, a dose dependent effect of *PNPLA3* G allele on the serum ALT activity has recently been described in a large study which included patients with chronic liver disease of various aetiologies [39]. When looking at our data, we realized that there is also a notable dose dependent effect of G allele in our cohort: the proportion of patients transplanted for CLF in the subgroups according to *PNPLA3* genotype increased with the number of G alleles (Fig 1B).

A relatively low number of subjects in the HCC group may be considered as the major disadvantage of our study. On the other hand, the comparison with a large number of population controls confirmed the same G allele frequency in the HCC group and population controls.

We also found that G allele carriers had a lower blood HCV viral load. This has been already known but it seems that the impact of the G allele on viral load is different in different HCV genotypes. Rembek et al. [40] reported a significantly lower viral load in GG homozygotes than in CG and CC genotype carriers infected with HCV genotype 2; however, the *PNPLA3* genotype had no impact on the viral load in subjects infected with HCV genotype 3. Contrarily, Eslam et al. [41] found no impact of the *PNPLA3* genotype on the viral load in a large study group, but the authors included subjects with various HCV genotypes (1–4) and they did not evaluate subjects with different genotypes separately. Our study group was homogenous regarding HCV genotypes: all patients were infected with genotype 1b and this fact allowed us to observe the impact of *PNPLA3* gene variants on the blood viral load. The HCV replication, virus assembly and release is linked to the host cell lipid metabolism. Endoplasmic reticulum–derived membranous web represents the viral RNA replication complex site and lipid droplets serve as virion assembly sites [42, 43]. It has recently been reported that HCV induces complex remodeling of the host cell lipid metabolism in order to enhance both virus replication and virions assembly [44]. The mechanism by which the PNPLA3 variant protein alters lipid turnover in hepatocytes has also been elucidated: the variant protein accumulates on the surface of lipid droplets [45] and binds the cofactor CGI-58 of adipose triglyceride lipase (ATGL or PNPLA2) [46]. Both inactivated ATGL and the barrier of PNPLA3 variant protein on the surface of lipid droplets impede lipolysis of triglycerides and their trafficking in hepatocytes. We assume that changes in lipid metabolism in hepatocytes caused by the PNPLA3 variant protein may affect the HCV life cycle. We consider the lower blood viral load in G allele carriers as a manifestation of the altered lipid trafficking in hepatocytes, but its impact on liver fibrosis progression remains unclear since long-term lowering of viral load by administration of low doses of interferon alpha had no beneficial effect on liver fibrosis progression in the HALT-C study [47].

## Conclusions

In conclusion, our results show that the pronounced liver steatosis and fibrosis in *PNPLA3* rs738409 G allele carriers with HCV genotype 1b cirrhosis may have a real impact on the timing and need of liver transplantation. The clinical consequence of G allele carriage could be a faster CLF development and need for liver transplantation at a younger age.

## Supporting information

S1 Table Patients’ clinical and laboratory data. (XLSX)

## Abbreviations

AFP: alpha-fetoprotein
ALT: alanine-aminotransferase
BMI: body mass index
CI: confidence interval
CLF: chronic liver failure
HCC: hepatocellular carcinoma
HCV: hepatitis C virus
HDL: high density lipoprotein cholesterol
INR: International normalized ratio
LDL: low density lipoprotein cholesterol
LT: liver transplantation
MELD: Model for End-Stage Liver Disease
NASH: non-alcoholic steatohepatitis
OR: odds ratio
PNPLA3: patatin-like phospholipase domain containing 3
SD: standard deviation
